# Conversations in Times of Isolation: Exploring Rural-Dwelling Older Adults’ Experiences of Isolation and Loneliness during the COVID-19 Pandemic in Manitoba, Canada

**DOI:** 10.3390/ijerph18063028

**Published:** 2021-03-15

**Authors:** Rachel V. Herron, Nancy E. G. Newall, Breanna C. Lawrence, Doug Ramsey, Candice M. Waddell, Jennifer Dauphinais

**Affiliations:** 1 Department of Geography and Environment, Brandon University, Brandon, MB R7A 6A9, Canada; 2 Department of Psychology, Brandon University, Brandon, MB R7A 6A9, Canada; newalln@brandonu.ca; 3 Educational Psychology, Brandon University, Brandon, MB R7A 6A9, Canada; lawrenceb@brandonu.ca; 4 Department of Rural Development, Brandon University, Brandon, MB R7A 6A9, Canada; ramsey@brandonu.ca; 5 Department of Psychiatric Nursing, Brandon University, Brandon, MB R7A 6A9, Canada; waddellc@brandonu.ca; 6 Centre for Critical Studies of Rural Mental Health, Brandon, MB R7A 6A9, Canada; dauphija37@brandonu.ca

**Keywords:** isolation, loneliness, aging, rural, pandemic, COVID-19, Canada

## Abstract

Older adults have been described as a vulnerable group in the current context of the COVID-19 pandemic. In Canada, where this study took place, older adults have been encouraged to self-isolate while the rest of the population has been cautioned against in-person contact with them. Prior to COVID-19, social isolation and loneliness among older adults was considered a serious public health concern. Using a series of semi-structured interviews with 26 community-dwelling older adults (65+) living in rural Manitoba, we explore older adults’ experiences of isolation and loneliness in the initial stages of the pandemic between the months of May and July 2020. Participants identified a loss of autonomy, loss of activities and social spaces (e.g., having coffee or eating out, volunteering, and going to church), and lack of meaningful connection at home as factors influencing their sense of isolation and loneliness. Although these loses initially influenced participants’ self-reported isolation and loneliness, the majority developed strategies to mitigate isolation and loneliness, such as drawing on past experiences of isolation, engaging in physically distanced visits, connecting remotely, and “keeping busy.” Our findings call attention to the role of different environments and resources in supporting older adults social and emotional wellbeing, particularly as they adapt to changes in social contact over time.

## 1. Introduction

In general, older adults are at a higher risk of contracting COVID-19, they are more likely to be hospitalized, and they are more likely to die from contracting the virus than other age groups [1]. As of July 2020, older adults (>60 yrs) represented 33% of the total cases, 70.2% of those hospitalized, and 96.7% of deaths due to COVID-19 in Canada where this study took place [2]. Initially, a high proportion of severe COVID-19 cases among older adults were linked to Long Term Care outbreaks in Canada [3]. Consequently, early in the pandemic governments identified older adults as a vulnerable group, instructing them to self-isolate, and encouraging greater caution and physical distancing from older adults as well as people with compromised immune systems [4]. The protective response toward older adults during COVID-19 raises questions about potential unintended consequences of physical distancing and isolation over time. 

Prior to COVID-19, academics and policy makers declared social isolation and loneliness among older adults a serious public health concern [5,6]. Studies have shown that prolonged social disconnection can put older adults at greater risk of various direct and indirect negative health effects, including influencing their immune system functioning, blood pressure, cardiovascular health, restorative sleep processes, and mental health outcomes such as depression and anxiety [7,8,9]. Holt-Lunstad, Smith and Layton [6] argue that social relationships not only influence quality of life among older adults, they also influence survival and isolation and loneliness should be taken as seriously as any other health risk factor. 

Social isolation—an objective lack of social contact or relationships with other people—is correlated but not the same as loneliness [10,11]. Loneliness refers to the gap between the quantity, quality, and mode of relationships one has and those they desire to meet their needs [12]. In analyzing these two concepts together, Newall and Menec [11] described four groups: the majority group (those not lonely nor isolated), the lonely in a crowd (lonely but not isolated), the life-long isolates, loners, or ‘lone farmers,’ (isolated but not lonely) and the vulnerable (both isolated and lonely). They argued that the latter three groups should not be assumed to be homogenous when considering their needs or designing interventions to support them. Indeed, older adults’ experiences, needs, and social contexts are diverse and dynamic. 

Older adults’ abilities to adapt to changes in social relationships are generally contingent on a range of mediating individual, social, and environmental factors [13,14]. Past studies on social isolation and loneliness among older adults have found individual level factors that can place people at risk of becoming isolated or lonely, such as demographic (e.g., age, marital status, income, gender, and level of education) and health characteristics (e.g., functional impairment and chronic conditions) [8]. For example, very old age, being single or living alone, and lower income can increase the likelihood of experiencing social isolation and loneliness [15,16]. In addition, there has been a growing interest in the role of environmental factors in experiences of isolation and loneliness [12]. 

Studies on environmental factors have focused on high levels of loneliness and isolation in deprived or socio-economically disadvantaged areas [17], as well as comparisons between urban and rural settings [18]. Quantitative studies comparing loneliness in urban versus rural settings reveal distinct factors influencing loneliness and isolation in rural areas as compared to urban areas [19]. Rural environments are characterized by greater physical isolation due to lower population densities, long distances to other centres, and lack of transportation and infrastructure that can contribute to isolation [20]. However, people living in rural communities tend to report a greater sense of community belonging than those in urban areas [21]. They may also develop and rely on a network of family, friends, and neighbours for support to a greater extent than their urban counterparts [22], but some research suggests that economic and social restructuring in rural places may leave older adults with much smaller support networks nearby [23,24]. Such local social and environmental conditions are perhaps more critical to understanding social isolation and loneliness, particularly during COVID times because of the restricted geographies that older adults and their social relations, younger or older, are living in today.

Contrary to common belief [25], the majority of older adults aged 65+ are highly socially engaged and have relatively high integration with a variety of social networks [16,26]. Indeed, older adults have had decades of experience in navigating multiple roles and relationships. However, the COVID-19 pandemic and associated physical distancing measures have brought tremendous change to the ways people typically engage with their social relationships and go about their day-to-day activities. Given that older adults have been identified as an at-risk group in the face of this pandemic, this may pose even more complexity in terms of how older adults maintain relationships during this time and balance their need to feel safe with their need to interact with others. We know little about older adults’ everyday experiences and place-based factors influencing levels of isolation and loneliness over the course of the pandemic. This study contributes a rich contextually-sensitive and temporal analysis of older rural adults’ experience before the pandemic, and several times during the pandemic.

It is critical that we examine how older adults are experiencing COVID-19 and public health measures, as the pandemic continues to disrupt social relationships and the ways we navigate our everyday social environments. The aim of our study was to investigate community-dwelling older adults’ experiences of physical distancing, isolation, and loneliness in rural communities in the Canadian province of Manitoba during the COVID-19 pandemic from May to July of 2020. We asked:

(1) How has COVID-19 influenced rural-dwelling older adults’ experiences of social isolation and loneliness during initial stages of the COVID-19 pandemic? 

(2) What factors and strategies influenced their sense of social isolation and loneliness over time?

### The Manitoba Context

On 20 March 2020, the Province of Manitoba declared a state of emergency and public health directives resulted in school closures, shuttering many community and non-profit services for seniors, and eliminating visits to older adults in long-term residential care [27]. All “non-essential” workers were encouraged to stay home, social gatherings outside the household were restricted, and the public were encouraged to keep six feet away from others in interactions with people outside their household. The federal government imposed the *Quarantine Act*, requiring those entering Canada to self-quarantine for 14 days. In addition, the Province of Manitoba initially required all out-of-province travelers to quarantine for 14 days. Initially, Manitoba contained the spread of COVID-19 in the spring and early summer of 2020, and most of the early cases were in the only census metropolitan area of the province, the city of Winnipeg. On 4 May 2020, the Province began their plan to “restore safe services.” All social gatherings (e.g., church, weddings, and funerals) were restricted to a maximum of 10 people and all food services remained closed, except for delivery, take-out and patio or walk-up food service. On 22 May 2020, public gatherings of 25 people were allowed indoors and 50 people outdoors and on 29 May 2020 outdoor visits to long-term care were allowed. Over the course of the summer the Province continued to reduce restrictions. However, by the end of July cases began to increase, including rural and small-town Manitoba. Our study focuses on participants’ experiences of initial restrictions and reopening and does not reflect older adults’ experiences as cases increased after July 2020.

## 2. Methods

To examine older adults’ experiences of physical distancing, isolation, and loneliness during COVID-19 we conducted semi-structured telephone interviews with people aged 65 years and older living in rural settings or small towns with less than 10,000 people [28]. The inclusion criteria were chosen to focus on a diverse group of older adults living in areas with fewer community resources and greater physical isolation. Semi-structured telephone interviews were used to collect in-depth reflections about participant experiences. Given the public health requirements to maintain physical distance, in-person interviews were not possible. Rather than using an on-line communication platform, we chose to conduct telephone interviews to make the research accessible to participants who may have less access to reliable internet services and limited digital literacy. 

### 2.1. Participants

With ethics approval from the University Ethics Committee, 26 participants were recruited through press releases, television and radio interviews, Facebook advertisements, and local active living centres in Manitoba. Participants’ ages ranged between 65 to 89 years. Most participants were female (*n* = 20) and retired (*n* = 22), although several participants were still working either part-time (*n* = 2) or full time (*n* = 2). Many participants had a high school (*n* = 7) or some college or university education (*n* = 14); three participants had less than a high school education and two participants held graduate degrees. One quarter of participants reported they lived alone.

### 2.2. Procedures

After obtaining oral consent, the principal investigator and two research assistants conducted 26 telephone interviews from May to June 2020. The interview began with a series of sociodemographic questions (e.g., when/where were you born; are you retired, what did you retire from; what is your highest level of education, and do you live alone?) and proceeded with more questions about their social networks, community, and activities during COVID-19 (e.g., Do you have any children or relatives living in your area? How many? How far away? How often do you normally see them? How many friends do you have in your community? How do you keep in touch with friends and family during this pandemic? What sorts of activities are you doing during this pandemic?). Participants were also asked to rate how often they felt lonely during a given week both prior to COVID-19 pandemic as well as currently (i.e., during COVID-19 pandemic) using a 4-point scale ranging from 1 (never, rarely, less than 1 day), 2 (sometimes, 1–2 days), 3 (occasionally, 3–4 days), and 4 (all the time, 5–7 days), a commonly used loneliness item taken from the CES-D scale [29]. Asking people to rate their loneliness in this way allowed us to examine whether people reported perceived changes (increases, decreases, no change) in their levels of loneliness pre-and during-COVID-19. At the end of the interview, participants were asked another series of open-ended questions about challenges, needs, and resources and strategies within their environments. All interviews were digitally recorded and ranged in length from one hour to an hour and 45 min.

From July to August, 25 participants took part in a follow up interview. These interviews were also semi-structured and repeated questions in relation to social isolation and loneliness. The open-ended questions focused on what, if anything, had changed for participants over the past month, including contact with family or friends, activities, and sense of community. Based on emergent themes in initial interviews, the guide also included a series of open-ended questions about what helped participants most during the COVID-19 pandemic and mandated physical distancing as well as what benefits and challenges they experienced living in a rural community during this time. Each follow up interview was digitally recorded and lasted between 30 min to an hour and a half.

Two interviewers participated in every interview, which supported data quality in multiple ways. One person took in-depth notes during the interview to support the audio-recording and enhance the quality of verbatim transcription. Post-interview, the two researchers discussed initial impressions and identified specific resource needs for participants who expressed need for support at the end of each interview. This allowed the researchers to refine their interview approach and ethical conduct around providing resources to participants as well as begin to develop some initial thematic areas of interest for analysis.

### 2.3. Analysis

First, analyses focused on the questions relating to participants’ reported isolation and loneliness before COVID-19, at the beginning of COVID-19, and one month later to begin to explore participants’ perceptions of how COVID-19 influenced their experiences of isolation and loneliness over time. We then used qualitative thematic analysis to examine what influenced participants’ isolation and sense of loneliness, as well as what conditions mitigated isolation and loneliness. Interview transcripts were coded and organized using Nvivo 12 software (QSR International, Melbourne, Australia). Three researchers coded the first transcript inductively with a focus on participant experiences. The primary researcher continued to code transcripts independently. On a weekly basis during data collection, the three researchers met to code a new transcript, review the code book, discuss divergent interpretations of the data, and come to a consensus on inductive codes. This process of regular initial coding meetings enhanced the credibility and confirmability of analysis by ensuring that interpretations were reviewed and confirmed by multiple researchers who were directly involved in the interviews. After a round of initial coding, the researchers grouped inductive themes under larger overarching categories in line with the research questions. Loss of autonomy and the loss of activities and social spaces were identified as dominant and distinctive challenges during COVID-19, while lack of meaningful connection at home was a significant factor influencing loneliness for the loneliest participants. Past experiences of isolation in rural places, negotiating physically distanced visits, connecting with others, and “keeping busy” were identified as resources and strategies to mitigate isolation and loneliness.

## 3. Results

**Figure 1:**
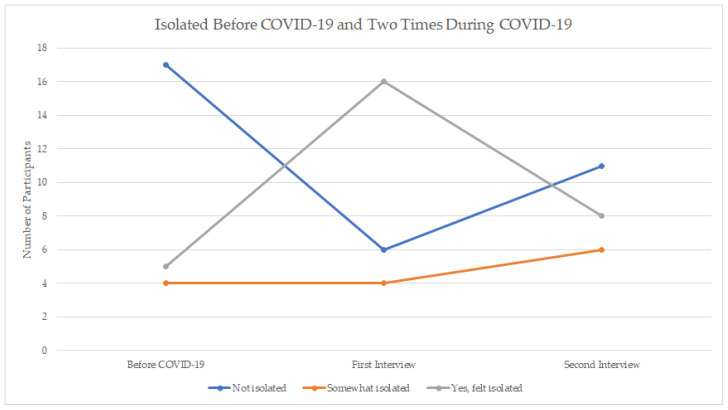
Changes in Social Isolation Before, Time 1 (May–-June), and Time 2 (July), by Participant.

Figure 1 shows the reported levels of isolation before COVID (retrospective), during the first interview, and during the second interview. Of the 17 people who reported NOT feeling isolated prior to COVID-19, all but four people reported being isolated during COVID-19 restrictions (May–June). Five of these 17 people had returned to feeling NOT isolated one month later; however, for the bulk of people (*n* = 12) the feeling of isolation persisted.

It is also of interest to consider those who felt isolated pre-COVID; 35% (9 people) reported feeling isolated before COVID-19. Two of these people reported feeling less isolated during their initial interview but had returned to feeling isolated one month later and three of these individuals, while they had reported continuing to feel isolated in their initial interview, reported being NOT isolated in their second interview. In other words, feelings of isolation appeared to have dissipated for a small number of people over the course of the study months.

Finally, we can also consider the four people who reported NOT feeling isolated prior to COVID-19 nor during COVID-19 (i.e., were NOT isolated at all 3 time points). It is particularly striking that this group of individuals did not feel isolated, given that Manitobans were facing restrictions on their day-to-day activities and contact with others.

**Figure 2:**
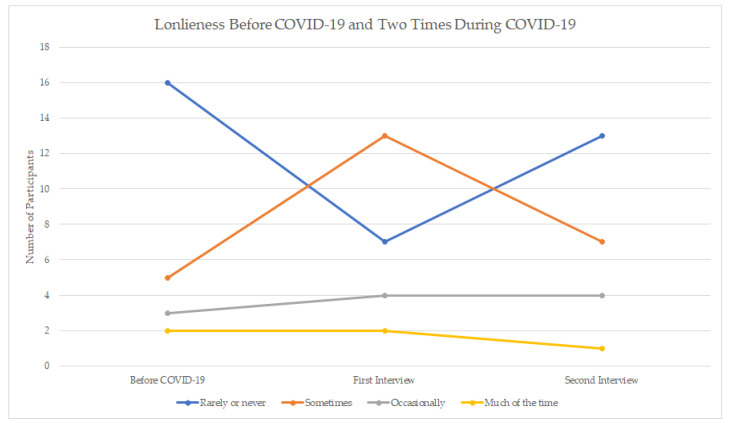
Changes in Loneliness Before, Time 1 (May–June), and Time 2 (July), by Participant. Sometimes (1–2 days/week); Occasionally (3–4 days/week); Much of the time (5–7 days/week).

Figure 2 shows the reported levels of loneliness at each time point. Results indicated that before the COVID-19 pandemic, most participants (61.5%) rarely or never felt lonely; and 19% felt lonely sometimes, 11.5% occasionally and 8% much of the time. When asked in the same interview about current loneliness levels in past week (during the months of May and early June), 27% felt rarely or never lonely and the majority (73%) felt lonely at least sometimes or more (Figure 2). A month later, at the second interview, 50% reported being rarely or never lonely and 50% reported feeling lonely at least sometimes or more. Therefore, as a group, one month later, people reported feeling less lonely than at the earlier time point in the pandemic.

It is of interest to examine further patterns over time. Of the 16 people who reported NOT feeling lonely pre-COVID, we can see that six people reported feeling lonely at the first interview during COVID-19 (May–June) but then returned to feeling NOT lonely by the second interview. However, seven people who had indicated being NOT lonely pre-COVID were lonely at the second interview, and thus had not gone back to pre-COVID levels. Only three people were consistently NOT lonely throughout all three time periods. Interestingly, four people who had reported feeling lonely pre-COVID ended up reporting feeling NOT (or rarely) lonely by the second interview. The qualitative results can highlight some of the nuances in the self-reported isolation and loneliness of participants.

### 3.1. Loss of Autonomy Due to COVID-19

Participants in the study identified loss of autonomy as a distinct factor influencing their experiences of isolation and loneliness during COVID-19. Joann (age 69) explained, “I feel isolated in the sense that I can’t do what I want to do when I, you know.” Similarly, Troy (age 70) said, “…it seemed forced. You know, uh, the COVID police are going to get you if you, uh, go out and do something. And, you know, again, I’m, I am pushing 70 pretty quick and it’s um, my immune system is compromised so, no, there is no chance.” In this case, loss of autonomy resulted from a combination of factors, including restrictions, individual health conditions, and perceptions of risk. Others explained that loss of autonomy was related to the shuttering of community spaces and restrictions on gatherings. As Kim (age 75) elaborated, “Well, living out here where I live, yes, I do feel isolated sometimes, yes. But I had a choice, if I didn’t want to feel isolated, I could go someplace or I could, you know, organize something, do something…That choice is not here now.” Similarly, Maria (age 70) explained, “… in the winter we sort of all batten down, you know, but we used to take little trips, we would go out to a restaurant, um, or whatever. So it was not as isolating.” Participants’ autonomy, choice, mobility and previous strategies for living in isolation were constrained during the initial phase of COVID-19. 

While many participants regained some autonomy and choice of activities by the time of the second interview, this was not the case for everyone, particularly older participants. One participant explained,

“I’m 88 years old and I don’t, when you get that old you’re not expected to last too much longer. And this here has taken away a good part of your last remaining time on earth. Because you’re, what you got pleasure out [of], you don’t get that anymore. So that’s taken away part of our life, that’s not that much of it left anymore. That’s one of the things that’s bothering us”

(George, 88)

George felt he had lost autonomy, and more poignantly, quality of life during the pandemic. He and some other participants continued to experience a loss of autonomy and social activities even as the province re-opened. The loss of autonomy and choice during COVID-19 was a factor that continued to influence some participants’ experiences of isolation and loneliness, as well as strategies for mitigating isolation and loneliness.

### 3.2. Loss of Social Activities and Spaces

Many participants observed that their increased isolation and sense of loneliness was influenced by a loss of everyday social activities and spaces. For example, Shirley (age 89) explained, “You can’t go to the neighbors for tea, the neighbor can’t come here. It was very isolating.” George (age 88) explained, “Well, you’re just bored with life. You just sit and argue with the wife. And that’s all the activities we have [soft laugh].” He described himself walking the halls of his apartment and looking out the window for something to do with his time. Although he was still able to go out to his daughter’s farm to garden, he described himself as bored and lonelier having lost other activities (e.g., volunteering and golfing) that filled his day.

Shuttering community spaces as well as familial pressure to isolate contributed to loss of social activities for many older adults. For example, Christopher (age 79) said, “I was playing cards about five times a week and now we don’t have cards at the [active living centre]. And the guy I was playing cards with in the lodge [congregate housing for older adults] here, he doesn’t want to play… His son phoned him from [country name] and told him he shouldn’t be playing cards.” In another example, Cheryl (age 75) explained, “the Legion of course is closed. We like to go to that Legion on Friday nights and socialize.” The participant noted how the closure influenced the routine interactions she enjoyed. She continued to explain that she volunteered to cook suppers at the Legion and she volunteered at the active living centre, both of which were closed. In describing their loss of social activities, almost half of participants mentioned lost volunteer opportunities, going to church, and coffee and eating out. Indeed, churches, coffee shops, restaurants, legions, and active living centres provided important everyday spaces for older rural-dwelling adults to get out of their homes and interact with others prior to the pandemic. 

At the time of the second interview, some participants regained familiar activities and spaces. For example, Jack (age 72) said “… Going to church, we started doing that again.” Although he did not go every week, he helped arrange cohorts to attend service in compliance with restrictions. Other participants commented how some spaces opened, but routines associated with those places changed: “we’re not allowed to have any cup of coffee or anything after church or barbeque even outside or any lunches… we’re sort of encouraged to go home as fast as we could” Cheryl (age 75). Moreover, social exchanges associated with familiar activities and spaces remained limited for most participants during the initial stages of COVID-19.

### 3.3. Lack of Meaningful Connection at Home

Some of the loneliest participants described specific circumstances in their home environment that contributed to their loneliness. For example. Helen (age 73), who felt extremely lonely at all three time points stated, “Yes. I feel isolated, alone, and lonely, a lot of the time… I got out more. Um, I don’t, I haven’t, I haven’t been able to get out very much because I can’t walk very far anymore.” Like other participants, she had lost places to go, but she also had mobility limitations that made her more confined to her home. In addition, she lived in a housing environment where she felt “…people in here don’t even talk to you [SCOFF], they just stay away from me, you know?” At the time of the second interview, she reported that her feelings and interactions had not changed. This example highlights the importance of environmental resources, like living arrangements and social relations within ones living environment, in mitigating loneliness.

In another example, Evelyn (age 71) stated, “I feel lonely quite often because I have lost my partner and my husband here. As dementia robs daily, and there is really no level of conversation at all.” Prior to COVID-19 her husband would walk to a drop-in and the post office in their community daily, while she had several coffee groups she attended throughout the week. Her husband still walked to the drop-in centre and post office and she explained to him daily why it was closed. Being at home caring for her husband living with dementia, Evelyn described herself as increasingly resentful. During the initial stages of the pandemic, she was diagnosed with anxiety and depression. Based on her first interview, the research team directed her to several Manitoba resources, including the daily hello program run by a non-profit organization, which connects older adults with one another for a regular telephone conversation. This participant reported that the daily hello program had helped her in the second interview. This was one reason she felt lonely occasionally in comparison to all the time, as she stated in her first interview.

### 3.4. Past Experiences of Isolation in Rural Places

Many participants in the study explained that they had some experience with isolation prior to COVID-19, which they could draw on during the pandemic. This corroborates the relatively high percentage of people reporting that they felt isolated prior to COVID-19 pandemic (Figure 1). For example, Arthur (age 71) said, “I enjoy being isolated.” Other participants preferred more social interaction but indicated that they had some experience with isolation. Jody (age 75) said, “I grew up on a farm… And we farmed for many years and that somehow prepares you for some isolation.” She also indicated that being at home with children was another experience of isolation she could draw from during COVID-19. Similarly, one participant explained that her recent health condition had forced her to isolate several years before COVID-19 and she felt more comfortable and safer at home: “Because of my condition, it hasn’t changed drastically. I don’t feel trapped. I feel safe and happy in our home. So being I learned that limit before” (Nora, age 73). Nora consistently reported being isolated, yet she also consistently reported low levels of loneliness before and during the pandemic. 

It is important to note that past experiences of isolation were not a positive resource for everyone. Several participants who had recently retired to the countryside, explained that they were isolated before COVID-19, but this was because of challenges related to integrating into a tightly knit community and lack of services and activities for older adults in the area. For example, Danielle (age 65) explained, “Rural Manitoba is, if you’re not born and raised in certain communities, it takes them a little bit of time to kind of include you.” Similarly, Amelia (age 69) explained how COVID-19 restrictions had not really changed their already isolated lives:

“First of all, social isolation that everybody has to go through now is basically our life. We’ve been here for four years, there’s not that many activities for seniors that we are aware of. We don’t go to church so there’s no church groups and stuff that we’ve been able to hook up with. So, there’s really nothing that we’ve been able to go to that we have chosen to go to.”

Both Danielle and Amelia identified being socially excluded because of the social dynamics and institutions associated with rural places as well as lack of services within their specific rural areas. For some isolated participants, there appeared to be a preference for isolation. However, for others, isolation appeared to be something they were ‘used to’ or perhaps even resigned to, with some attributing isolation to their rural context. It is interesting to note that past experiences of isolation appeared to help some participants cope during COVID-19.

### 3.5. Negotiating Physically Distanced Visits

Although participants experienced distinct losses and challenges during the initial stages of the pandemic, they also adapted and developed strategies to mitigate isolation and loneliness including physically distanced visits. Some participants engaged in outdoor visits. For example, Gerta (age 69) explained, “My friends and I are meeting at the end of the month, outside potluck at my friends place, and we will all do our social distancing, um, so that’s, we’re trying for that and we will continue.” Although outdoor visits increased during the summer, participants were selective in the way they visited and with whom they visited. Nora (age 73) stated, “We haven’t allowed anybody into our house and not even for washroom privileges.” A small number of participants also had indoor visits. Joann (age 69) said, “our bubble is getting bigger. Because the numbers—we don’t have any, any cases in our region at this time… we actually had a couple other relatives sit inside to have dinner with us, as it was cold outside. So that’s something that we would not have done prior to this, prior to the month.” Participants explained a range of contingencies influencing how and where they visited including home ownership, whether they had access to outdoor space, their health condition and the health condition of their social relations, and perspectives of their social relations. 

Others commented on how their social network either wanted to protect *them* by not visiting or on how they themselves did not want to put some of their more vulnerable contacts at risk. In other words, interestingly, participants described *seeing others* as at risk or *being seen* as an at-risk group themselves. In her second interview, Cheryl (age 75) commented “I’m a little let down that you know some of the families that, uh, with children that we normally would have seen, umm, aren’t too interested in coming near us” and another participant commented,

“I still don’t feel comfortable doing that [visiting] with some of the other people I know because they got diabetes, or they got, uh, a slow-moving form of cancer or something like that, so, you know, it’s expanded a little but, I am getting to the point where I am missing, um, interacting with people”.

(Maria, age 70, interview two)

These comments reveal the increased difficulty of navigating social relationships, specifically co-presence (engaging in real-time face-to-face interaction), even if it is physically distanced.

### 3.6. Connecting during COVID-19

Most participants developed strategies for remote connection with others, by phone, text messages, emails, video calls, and social media. For example, Jody (age 75) remarked, “Ah, Facebook is a wonderful thing to keep track of people a little bit and be able to communicate. Ah, if you so desired, the phone is wonderful and so is the computer…” She explained that she had planned to call someone every day, but she found she was too busy with other activities. One quarter of participants described developing routine check-ins with friends, family, or neighbours. For example, in their second interview, George (age 88) explained, “Well, [soft laugh], that’s kinda what keeps us going. The wife, she is happy talking on the phone for several times a day with the family. And they’re checking in on us and we are seeing how things are going with them...” Therefore, participants utilized a variety of means of communicating remotely with each other using telephone or other technologies.

Some other participants revealed the limitations of remote connection in terms of meeting ones need for physical touch. Ava (age 80) explained she had regular phone and text conversations with friends and family. She said: “I’m managing fine, you know, when I’m used to having people, it was, it was lonely. And yet, I mean I can’t complain because people do phone and that, but it’s not the same as, like even my daughter, I couldn’t go hug her, you know.” Other participants talked about how much they looked forward to hugging friends at some point in the future, highlighting the importance of physical contact.

A smaller number of participants identified a loss of communication during COVID-19, with some of this loss due to limitations of technologies in rural areas. Danielle (age 65) lived in an area without cell service and internet. She explained, “Like you text people now, nobody really answers the phone.” She felt isolated by a lack of infrastructure but also much broader societal changes in how people communicate. Audrey (age 80) commented that even though she continued to contribute to the community she missed the communication and social interactions that used to come with these activities. Of these community efforts she said, “So, you know, it was an isolation. And nobody could come here. I was a drop off place for a gal who sold head bands and masks for the hospital. And so, she would put them in my mailbox outside, and you know, we did not even talk.” Although she continued to connect with community members through volunteer work, it did not meet her social needs in the way it had in the past. Other participants waiting for medical procedures also identified a loss of communication with the hospital that contributed to their experience of isolation during COVID-19. For example, Maria (age 70) explained, “Right now, I feel extremely isolated. I am waiting for gallbladder surgery. I haven’t had any notification from the hospital or the health region, I’m in a lot of pain.” Not knowing what was happening and trying to keep herself in strict isolation to be ready for surgery, Maria felt very isolated in relation to a lack of communication and clear information.

### 3.7. “Keeping Busy”

Most participants identified “keeping busy” or “filling the day” with household chores, hobbies, getting outside, walking, and gardening, as strategies for mitigating isolation and loneliness. In the face of a loss of activities and social spaces, many participants adapted their activities and the environments in which they engaged in routine activities. Alice (age 75) explained, “There were days where I did not even talk to anybody. I talked to my cat [laughing]… You know what saved me was I did exercises on a YouTube video on my TV.” She missed going to dancing classes and searched for activities to substitute her pre-COVID activities. At the time of the second interview, some participants experienced greater freedom and opportunities to “keep busy.” For example, Evelyn (age 71) explained

“I would say I feel a little bit more free, like, than I was. Because I was ordering in my groceries and things like that to start out with. But now I am going into the store and buying my own stuff. Um, going into the hardware store, getting what I need. Fixing, you know, fixing the things that I need to fix. And so, I don’t feel as isolated because I’m interacting.”

For this participant who was very isolated and lonely in the first interview, the ability to get out for such small tasks was particularly important. In another example, George (age 88) explained how walking helped fill his days: “instead of going to visit people, if you see one on the streets you wave at him and say hi and keep going.” Many of the things people chose to do to fill their days were independent activities that took them out of the house and enabled brief encounters with others walking on the street or in the grocery store.

## 4. Discussion

The COVID-19 pandemic has changed our lives in many ways and continues to have tremendous implications for how we navigate our social environments. For the present study, we had the opportunity to have in-depth discussions with a small group of rural-living older adults over the course of three months during May–July 2020 in Manitoba. Although three months is a brief period, significant changes took place during this time. The longitudinal patterns in self-reported isolation and loneliness we observed suggest that, as time went on and restrictions eased, many older adults were feeling less socially isolated and lonely and were possibly returning to pre-COVID levels. We explored pre-COVID-19 levels of isolation and loneliness as well as factors that lessened or contributed to loneliness and isolation during pandemic restrictions.

Notably, in this sample of rural community-dwelling older adults, pre-COVID levels of perceived isolation and loneliness were quite high. For example, the present study sample in which almost 20% reported being occasionally (or more) lonely pre-COVID can be compared with 11% of adults ages 65+ reporting this level of loneliness in a Canadian population study [30]. Although a small number of participants suggested a preference for isolation, other participants pointed to a lack of programs, services, and social connections in their rural environment as factors contributing to their isolation prior to COVID-19. This is consistent with the broader research literature on age-related social exclusion of older rural adults [31], and more generally, on rural-urban differences in older Canadian adults’ health and well-being [32]. Although older adults in rural areas may have been accustomed to a greater degree of social isolation, not surprisingly, most participants in the study reported feeling *more* isolated because of the profound physical distancing required with the COVID-19 restrictions. One of the unique contributions of this study, is that some older adults identified past experiences of isolation in rural places as a resource for coping with isolation during COVID-19. Like many of the other strategies identified, drawing on past experience was not without limitations, but some older adults suggested it helped them cope. 

Participants explained initial changes as a consequence of losing autonomy and choice as well as social activities and spaces that enabled them to mitigate isolation and loneliness in the past. Participants highlighted the importance of volunteering, going to church, and having coffee or eating out as protective strategies prior to COVID-19. The significant role of volunteering in the lives of study participants is consistent with research on voluntarism within rural communities, which suggests that involvement and opportunities to engage in local volunteer activities can mediate feelings of isolation and loneliness [33]. Moreover, many participants explained the importance of routine social spaces and interactions in their communities in pre-COVID times, consistent with geographical research on aging in place [34]. As restrictions changed, some participants regained access to these spaces but routines in relation to face-to-face social contact had changed. Although participants acknowledged the loss of social space as a challenge, many adapted and developed different strategies for meeting their social needs in their changing environments.

Some participants met their social needs through outdoor or physically distanced visits. It is important to highlight that this research took place in the summer months when weather was more conducive to outdoor visits. Several other factors influenced older adults’ in-person visits including their age, whether they owned their own home or lived in a congregate environment, whether they had access to outdoor space, whether they had neighbours close by, access to transportation and mobility limitations, their health status, the health status of others within their social networks, the number COVID-19 cases in their region, and the perspectives of their social relations. The most consistently lonely participants (those who reported being lonely most of the time) lacked access to many of these resources (e.g., they could not drive, did not own their own home, and had pre-existing health conditions), highlighting the role of social and economic inequalities in contributing to isolation and loneliness before and during COVID-19 [17,35]. In addition, they were more confined to their homes (e.g., experienced mobility limitations or had care responsibilities) and lacked meaningful connection within their homes. 

Participants found ways to connect remotely through phone and video calls, text messages, emails, and social media. Consistent with existing research, some older adults lacked access to telecommunications infrastructure or digital literacy skills to make use of cell or internet services, which influenced their ability to connect face-to-face at a distance [36,37]. Such gaps in infrastructure and literacy are critical considerations for future interventions particularly in relation to rural environments. Although COVID-19 expanded access to resources for some older adults as they found more activities online, this was certainly not true for all. Participants also noted changes in routine communication with community members and the healthcare system itself, which influenced their sense of isolation and loneliness. Finally, while many participants replaced physical contact with remote contact, they suggested that remote contact failed to meet their needs for physical touch. 

Our study highlights the continued importance of paying attention to the environments and resources surrounding older adults and their influence on isolation and loneliness. Participants who felt the loneliest revealed the importance of resources and strategies for mitigating loneliness in their homes and surrounding communities, including the need for meaningful conversations during times of isolation. This was particularly the case for an isolated dementia carer, in the study, calling attention to the importance of remotely delivered social programs. Indeed, participants’ narratives suggest that both before, and during COVID-19, it was important to have a range of choices, activities, and places to go; this is not surprising, but the challenge ahead lies in how local communities and organizations support initiatives that meet the social needs of older adults safely.

Local communities and organizations can learn from the challenges older adults faced and strategies they developed. For example, they can promote the development of modified, flexible or new activities such as online or physically distanced programs and services. The older adults in the study identified limitations of online and remote initiatives, but they also identified low tech strategies for mitigating isolation and loneliness such as telephone check-ins, using telephone support services, physically distanced outdoor visits, and keeping busy (e.g., walking and gardening). Some of these strategies can be supported at the community level through campaigns to end loneliness, continuing safe volunteer opportunities for older adults, as well as access to safe walking paths and other outdoor community spaces. 

## 5. Limitations

The early results of this study paint a somewhat hopeful picture—many older adults adapted and, among those who experienced increased isolation and loneliness, many returned to pre-COVID levels of isolation and loneliness—but the pandemic continues. We do not know how older adults’ experiences have changed even six months later. Continued longitudinal work on the impacts of the pandemic is critical to understanding the long-term impacts on social isolation and loneliness. We acknowledge that the three-month window in which data collection took place for this study was a small window of time, yet it was also a time of tremendous change and uncertainty in the lives of older adults. Capturing older adults’ perceptions of this change, through a rating scale and subsequent explanation, as well as their strategies for responding to significant change is important to inform public health policy and practice. Public health policy and practice that is out of touch with, or insensitive to, perceptions and experiences is likely to be ineffective or may even be harmful. Another limitation of our research is that most participants lived with someone and more women than men participated in interviews. In addition, few participants self-identified as racialized or Indigenous. Future research should explore the specific experiences of those who live alone as well as the gendered, classed, and racialized experienced of social isolation and loneliness during this time. Despite these limitations, the strength of this study involved its emphasis on capturing rich descriptions of this very particular time and place, characterized by unprecedented isolation. Future qualitative research should continue to explore older adults’ experiences and responses to change during the COVID-19 pandemic and beyond, with particular attention to their environments and resources.

## 6. Conclusions

Rural community-dwelling older adults are a diverse group with different resources and strategies influencing their pandemic experiences of social isolation and loneliness; however, they share experiences of living in isolated places prior to COVID-19. Consequently, many had developed strategies to manage isolation and protect themselves from loneliness prior to the pandemic, some had resigned to a degree of isolation attributing it to their rural context, and a smaller number identified as isolates by preference, perhaps fitting into the ‘lifelong isolates’ grouping discussed by Newall and Menec [11]. This is not to suggest they were unaffected or that they did not need resources to mitigate isolation and loneliness. On the contrary, the pandemic and associated restrictions have reinforced the importance of autonomy and choice, as well as access to everyday social spaces and activities within ones’ immediate environment. Even as restrictions eased, not all participants regained autonomy and choice and many of the routines associated with social spaces changed. In addition, participants experienced new challenges as they negotiated physically distanced visits. Using communications technology, checking in with others, and engaging in a range of activities to keep busy were helpful for older adults in this study. However, these strategies were not without limitations and they were not universally accessible. Moreover, the pandemic highlights the continued importance of place-based resources for protecting against isolation and loneliness. As the pandemic continues it is essential that older adults continue to have choices, places to go, people to engage with, and a range of activities within their homes and their surrounding communities. 

## Data Availability

The data reported were not publicly archived.
